# The Effectiveness of Circumferential and Sectional Matrix Systems in Obtaining Optimum Proximal Contact in Class II Composite Restorations: A Systematic Review

**DOI:** 10.7759/cureus.84967

**Published:** 2025-05-28

**Authors:** Shamal Kamble, Manoj Ramugade, Abrar Sayed, Kishore Sapkale, Arti Gulhane, Aditi Magar

**Affiliations:** 1 Conservative Dentistry and Endodontics, Government Dental College and Hospital, Mumbai, Mumbai, IND

**Keywords:** circumferential matrix band system, class ii cavity, class ii composite restoration, composite filling, composite restoration, conservative dentistry and endodontics, dental cavities, matrix band system, proximal contact, sectional matrix band system

## Abstract

This review aims to evaluate the effectiveness of circumferential matrix band (CMB) and sectional matrix band (SMB) systems in obtaining optimum proximal contact in class II composite restorations.

This review was performed according to the Preferred Reporting Items for Systematic Reviews and Meta-Analyses (PRISMA) 2020 guidelines and registered in PROSPERO (CRD42024556368). Electronic databases were searched from January 1990 to April 2024 for studies assessing the effectiveness of circumferential and sectional matrix systems in obtaining optimum proximal contact in class II composite restorations. Quality assessment or risk of bias assessment of included studies was performed using the Cochrane Risk of Bias (RoB) 2 tool for randomized controlled trials (RCTs) through its domains using Review Manager (RevMan) software version 5.3 (The Cochrane Collaboration, London, UK).

Six studies fulfilled the eligibility criteria and were included in the qualitative synthesis. Quality assessment revealed a presence of moderate to low risk of bias. It was observed that sectional matrix band systems were superior and provided better results as compared to circumferential matrix band systems with regard to the parameters assessed.

This study found the sectional matrix band system to be more effective than the circumferential system in achieving optimal proximal contact in class II posterior composite restorations. Sectional matrices with separation rings produced significantly tighter contacts. Although operator satisfaction was similar for both systems, the sectional matrix was deemed easier to use. Overall, the sectional matrix system is preferred for achieving stronger, more consistent proximal contacts in clinical settings.

## Introduction and background

Since its existence, mankind has been affected by various diseases, including oral and dental conditions, with dental caries being the most prevalent. Dental caries are mainly categorized into pit and fissure caries and smooth surface caries. Among smooth surface caries, class II lesions are the most frequent, as the interdental space naturally fosters bacterial colonization and poses challenges in maintaining effective cleanliness [1].

Caries progression pattern in smooth surface caries impacts the wider area of proximal contact, leading to more damage in the proximal contact area [2]. When caries disrupts the natural tooth contacts, it can lead to several issues, including periodontal diseases, tooth shifting, food impaction, and compromised dental arch stability. Therefore, restoring proper proximal contact and contour is crucial for maintaining an optimal stomatognathic system and achieving a well-balanced functional occlusion [3].

Among all smooth surface carious lesions, class II cavities require adequate clinical skills and knowledge, and achieving ideal proximal contact is a known challenge in class II direct composite restorations. Various restorative techniques and matrix systems have been proposed to overcome these restoration challenges to produce natural contours and embrasures [4].

Establishing proximal contact areas relies heavily on matricing and tooth separation techniques. Various systems of matrix bands have been utilized to restore cavities with missing walls. For class II cavity restorations, the circumferential matrix band (CMB) system has traditionally been favored owing to its stability and user-friendly nature. In traditional circumferential matrix systems, such as with stainless steel bands, the thickness ranges from 0.038 mm to 0.05 mm. This system is particularly recommended for cases involving missing adjacent teeth or misaligned dentitions. However, a circumferential matrix band system is not without its drawbacks. These include poor adaptation to neighboring teeth, challenges in recreating natural anatomical contours, and an increased likelihood of developing marginal overhangs [5,6]. Over time, the matrix band system evolved, and newer systems are designed to be less challenging to use and to compensate for the shortcomings and complexity of the earlier versions [7].

Although the sectional matrix band (SMB) system has drawbacks, such as technique sensitivity, concave contact formation, and placement distortion, it is a relatively recent innovation. It has been shown to create tight proximal contact points (PCPs), facilitate faster tooth separation, and enhance the anatomical emergence [8].

Currently, there is a lack of comprehensive research that combines qualitative and quantitative analyses to compare the efficacy of circumferential and sectional matrix systems in establishing the optimal proximal contact for class II composite restorations. Therefore, this systematic review aimed to assess and compare the performance of these matrix systems in restoring appropriate proximal contact in class II composite restorations.

## Review

Materials and methods

This systematic review was performed in compliance with the Preferred Reporting Items for Systematic Reviews and Meta-Analyses (PRISMA) 2020 guidelines. It was also documented in the PROSPERO database (Prospective Registration of Systematic Reviews) under the identification number CRD42024556368.

The study was structured using the PICO format, with the following criteria: population, teeth affected by proximal caries, intervention, circumferential matrix system, comparison, sectional matrix system, outcome, clinical effectiveness in achieving proximal contact, study design, randomized controlled trials (RCTs), clinical trials, and in vivo studies.

The inclusion criteria were studies that evaluated posterior teeth with proximal caries, comparative analyses of circumferential and sectional matrix systems, randomized controlled trials, clinical trials, in vivo publications from January 1990 to April 2024, and studies written in English. The exclusion criteria comprised studies centered on non-interproximal cavities and comparisons involving alternative matrix systems, as well as literature reviews and abstracts, letters to the editor, editorials, in vitro studies, case series, case reports, and animal studies.

Search Strategy

A thorough electronic search was performed from January 1990 to April 2024 using PubMed, Google Scholar, and cross-referencing. Additionally, a manual search was conducted in endodontic journals, including the International Endodontic Journal, Journal of Endodontics, Saudi Endodontic Journal, and Journal of Conservative Dentistry. The search was refined using keywords and Medical Subject Headings (MeSH) terms combined with Boolean operators (AND/OR): ("Matrix Band"[MeSH] OR "matrix system" OR "sectional matrix" OR "circumferential matrix") AND ("Composite Resins"[MeSH] OR "composite restoration" OR "resin restoration" OR "Class II restoration") AND ("Dental Contacts"[MeSH] OR "proximal contact" OR "contact point" OR "proximal contact tightness" OR "contact quality") AND ("Effectiveness" OR "clinical outcome" OR "contact accuracy" OR "restoration quality").

Screening Process

Search and screening were performed by two independent investigators following a two-phase selection process. In the first phase, titles and abstracts were reviewed, and studies that did not meet the inclusion criteria were excluded. In the second phase, full-text articles were independently assessed. Corresponding authors were contacted for additional information when necessary.

Study Selection

**Figure 1:**
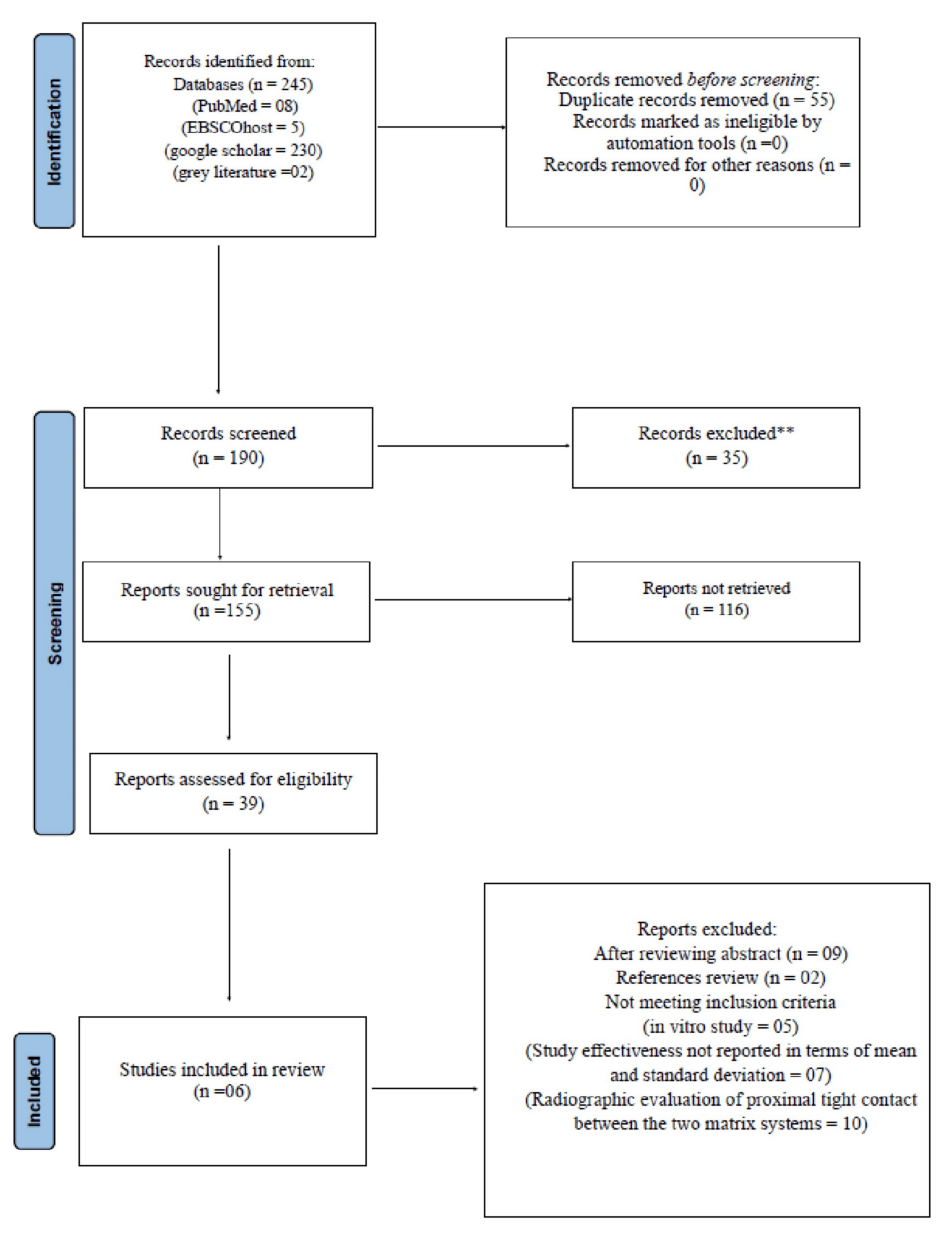
PRISMA flowchart PRISMA: Preferred Reporting Items for Systematic Reviews and Meta-Analyses

After duplicate removal and screening of reference lists, 116 studies were excluded. The remaining full-text articles were assessed for eligibility, and six studies that met the inclusion criteria were included in the qualitative synthesis (Figure 1).

Data Extraction

Two independent reviewers extracted descriptive details from all included studies using customized data extraction forms in Microsoft Excel. The forms contained headings for author(s), country of study, year of study, sample size, study design, outcome assessed, and conclusion.

Assessment of Study Quality

The Cochrane Risk of Bias (RoB) 2 tool was employed to evaluate the methodological quality of the included studies. This tool examines various domains, including random sequence generation, allocation concealment, blinding of personnel and equipment, blinding of outcome assessment, incomplete outcome data, selective reporting, and other biases. The assessment was conducted using Review Manager (RevMan) software version 5.3 (The Cochrane Collaboration, London, UK). Based on the domains and criteria, individual studies were classified as having low, moderate, or high overall risk. A study was deemed low risk only if all domains were assessed as low risk. High risk was assigned if one or more domains were found to be at high risk. Moderate risk was given to studies with one or more uncertain domains and no high-risk domains.

Data Analysis

For continuous outcomes, the standardized mean difference (SDM) with 95% confidence interval (CI) was computed. When heterogeneity was absent (p > 0.05 or I-squared ≤ 24%), a fixed effects model (Mantel-Haenszel method) was employed; otherwise, a random effects model (DerSimonian-Laird method) was utilized. RevMan software version 5.3 (The Cochrane Collaboration, London, UK) was used for all statistical analyses. Statistical significance was determined at p < 0.05.

Evaluation of Methodological Quality in Included Studies

**Figure 2:**
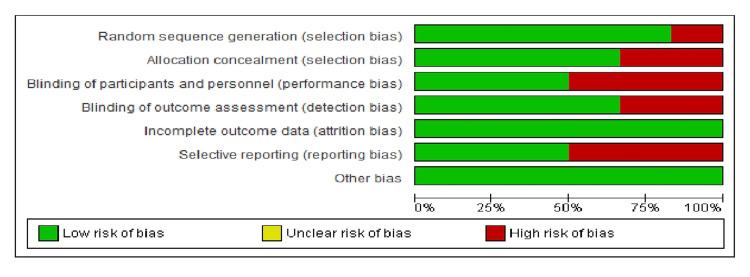
Risk of bias graph: review of authors' judgements on each risk of bias item presented as percentages across all included studies

**Figure 3:**
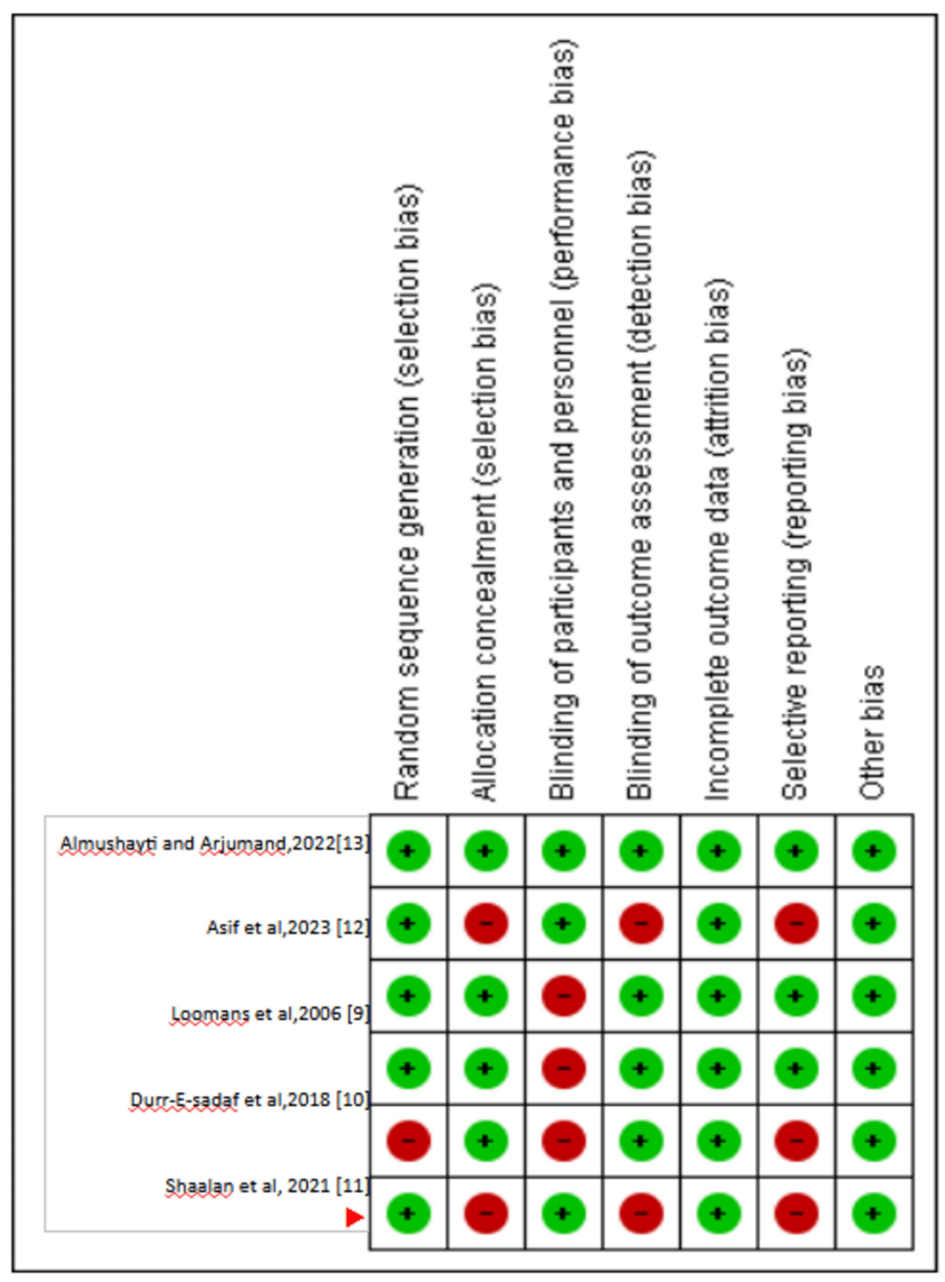
Risk of bias graph

Figure 2 and Figure 3 illustrate the risk of bias in the included studies as assessed through the Cochrane Risk of Bias (RoB) 2 tool [9-13]. The methodological quality of all included studies was largely comparable. Each study exhibited a moderate to high risk of bias across all relevant domains. The highest risk of bias was observed in random sequence generation (selection bias), blinding of participants and personnel (performance bias), blinding of outcome assessment (detection bias), incomplete outcome data (attrition bias), and selective reporting (reporting bias). Among the studies, Durr-E-Sadaf et al. (2018) [10] and Shaalan et al. (2021) [11] demonstrated the highest risk of bias compared to others. Asif et al. [12] showed minimal risk of bias. Almushayti and Arjumand (2021) [13] showed the lowest risk of bias. The allocation concealment (selection bias) and other bias domains were assigned the lowest risk.

Results

As presented in Table 1, data from six studies of the 245 studies screened were analyzed, encompassing a total of 1,720 class II cavities requiring composite restorations, where the effectiveness of sectional and circumferential matrix band systems in achieving optimal proximal contact was assessed. All included studies followed a randomized clinical trial (RCT) design. Geographically, two studies were conducted in Pakistan, two in Saudi Arabia, and two in Egypt. Each study evaluated the ability of both matrix systems to achieve better proximal contact. The findings indicated that sectional matrix band systems were generally superior, providing better outcomes compared to circumferential matrix band systems in most studies.

**Table 1:** Descriptive characteristics of the included studies RCT: randomized controlled trial

Journal name	Author and publication year	Study design	Parameters assessed	Experimental group	Control group	Outcome evaluated	Summary
Journal of Dentistry	Wirsching et al. (2011) [5]	RCT	Effect of two matrix systems on the proximal contact tightness of direct posterior composite restorations	Circumferential matrix system	Sectional matrix system	To investigate the influence of cavity preparation (MO/DO/MOD) and type of matrix system on the proximal contact tightness of direct posterior composite restorations	The use of the sectional matrix system in two-surface class II cavities resulted in statistically significantly tighter proximal contacts than the use of the circumferential matrix system.
Journal of Dentistry	Loomans et al. (2006) [9]	RCT	Clinical changes in proximal contact strength using a circumferential and sectional matrix system with separation rings	Circumferential matrix system	Sectional matrix system	Clinical changes in proximal contact strength after inserting class II composite resin restorations	Class II posterior composite resin restorations placed with a combination of sectional matrices and separation rings resulted in a stronger proximal contact than when a circumferential matrix system was used.
Indian Journal of Dental Research	Durr-E-Sadaf et al. (2018)[10]	RCT	Effect of matrix system on the proximal contact points and contours in posterior teeth with class II cavities	Circumferential matrix system	Sectional matrix system	To compare the effects of two matrix band systems, the circumferential matrix system and the sectional matrix system	The sectional matrix band system has been found to be superior to the circumferential matrix band system.
Journal of International Oral Health	Shaalan et al. (2021) [11]	RCT	Use of two different matrix systems for the reproduction of proximal contact	Circumferential matrix system	Sectional matrix system	To assess the influence of different matrixing techniques, either a sectional matrix or a circumferential matrix, on obtaining proper proximal contacts	Optimum contact points were highly associated with the sectional matrix system.
Journal of Ayub Medical College Abbottabad	Asif et al. (2023) [12]	RCT	Contact tightness	Circumferential matrix system	Sectional matrix system	To compare the contact tightness achieved with two matrix band systems	The sectional matrix band system was statistically superior to the circumferential matrix band system.
Cureus	Almushayti and Arjumand (2022) [13]	RCT	Operators' comfort and satisfaction were evaluated according to their assessment of the contact points they reproduced and the emergence profiles of restorations, using a circumferential matrix system and a sectional matrix system	Circumferential matrix system	Sectional matrix system	To investigate the operators' ease, satisfaction, and comfort in using a circumferential matrix system and a sectional matrix system on the proximal contact points	Circumferential and sectional matrix band systems showed no significant differences in operators' satisfaction, but it was easier to use the sectional matrix band system than the circumferential matrix band system.

Discussion

Proximal contact is a dynamic physiological feature influenced by multiple factors, including tooth type, position, mastication, and restorative procedures. It plays a crucial role in preserving and stabilizing the dental arch. The absence of proximal contact can result in food impaction, periodontal diseases, proximal caries, tooth displacement, and, ultimately, loss of dental arch integrity [9,10].

There are several matrix systems available on the market that are specifically intended for posterior direct composite restorations. The two most frequent ones are the circumferential matrix band (CMB) and sectional matrix band (SMB) systems [11]. Circumferential matrix systems, such as the Tofflemire system, were first introduced in 1946 by Dr. Joseph Tofflemire [12]. Circumferential matrix systems are easy to use and minimize time, but they can replicate a single point of contact rather than an entire area [13]. To address these issues, traditional matrix systems were transformed into a novel sectional matrix system for the restoration of class II composite resin. To achieve proximal contact, a matrix band is used to restore the contact area. Its primary function is to replace the missing wall, contain excess restorative material, and simplify the process of restoring the proximal contact area [14,15].

Loomans et al. conducted a randomized controlled trial to examine the proximal contact of posterior composites utilizing CMB (with wooden wedge and hand instrument) and SMB (separation ring), with contralateral teeth serving as a control. A tooth pressure meter was used to conduct the evaluation. They discovered that class II posterior composite restorations applied with a mix of section matrices and separation rings provided better proximal contact than a circumferential matrix system. This result was attributable to the stronger separating effect of the ring when compared to the usage of hand instruments [9].

Wirsching et al. (2011) also conducted a randomized controlled trial to evaluate the tightness of proximal contacts in posterior composite restorations involving two and three surfaces. They utilized a tooth pressure meter device for their assessment. Their findings revealed that in two-surface class II cavities, the use of a separating ring led to tighter proximal contacts. However, when examining three-surface cavities, the study found no statistically significant difference between the two matrix systems employed. This is due to the fact that the two rings and matrices arranged simultaneously both medially and distally provide a separation effect in the opposite direction, which reduces their effect in the contact area [5]. Durr-E-Sadaf et al. (2018) [10] evaluated the effectiveness of CMB and SMB in repairing two-surface cavities in posterior teeth. The proximal contact point (PCP) was measured by passing a dental floss. They discovered that CMB had more overhanging proximal borders and defective contact points than SMB. Despite higher results with SMB, a considerable majority of students favored CMB due to its ease of use and the lack of training required for SMB [16].

Shaalan et al. (2021) [11] discovered that regardless of the operator's experience, optimum contact sites were more closely connected with SMB than CMB. They discovered that pre-contoured section matrices with an interdental separation ring caused a significant increase in total contact tightness, whereas the flat CMB produced a significant decrease in contact tightness. As a result, the thickness and shape of the matrix band may influence the contact tightness. Similarly, inadequate separation caused by wedge insertion could be the etiology of the open contact in CMB [17].

The aim of the study by Almushayti and Arjumand [13] was to analyze the ease with which operators could use matrix band devices to restore class II cavities. Of the students, 48.6% believed that CMB's difficulty stemmed from the need for extra time for placement. Additionally, 57.1% of students thought that the difficulty of SMB stemmed from a lack of training or expertise. The study's overall outcome was that the ease and convenience of using both systems do not differ statistically significantly [18].

Asif et al. (2023) conducted a randomized controlled trial to compare the contact tightness of CMB (Tofflemire) and SMB (Palodent) for class II composite repair. The evaluation was performed with dental floss and the FDI World Dental Federation clinical rating standards of contacts. They discovered that the SMB Palodent contact outperformed the Tofflemire matrix method solely in men. There was no statistically significant difference between the two systems in women. Male teeth are often larger than female teeth, which explains this. This implies that the contact point is generally larger and more difficult to construct [12].

A review of the existing information reveals a severe dearth of research comparing sectional and circumferential matrix band systems in terms of clinical outcomes. Although a comprehensive and unrestricted search was conducted based on the established eligibility criteria, only a limited number of studies qualified for qualitative analysis, with just six being included in this systematic review. To develop a more robust and evidence-based repository, it is strongly recommended that well-designed randomized controlled trials and clinical studies be undertaken.

Limitations

The study had a limited sample size, which may affect the generalizability of the results. Additionally, variability in operator experience and technique could have influenced the outcomes. The scarcity of randomized controlled trials on this topic highlights the need for further high-quality research to confirm these findings. Future studies should incorporate larger sample sizes, diverse clinical settings, and long-term follow-ups to provide more comprehensive evidence on the effectiveness of sectional and circumferential matrix band systems in restorative dentistry.

## Conclusions

The findings of this study indicate that the sectional matrix band system is superior to the circumferential matrix band system in achieving optimal proximal contact in class II posterior composite resin restorations. The use of sectional matrices combined with separation rings resulted in significantly tighter proximal contacts compared to the circumferential matrix system. Additionally, while no significant differences were observed in operator satisfaction between the two systems, the sectional matrix band system was considered easier to use. Given these advantages, the sectional matrix system appears to be the preferred choice for achieving stronger and more consistent proximal contacts in clinical practice.

However, an important limitation in the reviewed studies is the lack of consideration for operator competency and experience, which could have influenced clinical outcomes, especially given the technique-sensitive nature of matrix placement. Furthermore, this review focused solely on traditional circumferential matrices and did not account for recently introduced thin, pre-contoured anatomical circumferential matrix bands, which may offer improved adaptation and contact formation. Future studies should consider evaluating these modern anatomical circumferential matrices in comparison to both traditional circumferential and sectional systems to better understand their clinical effectiveness in achieving optimal proximal contacts.
